# Work engagement, job satisfaction and turnover intention among nurses working in private hospitals of Pokhara, Nepal

**DOI:** 10.1371/journal.pone.0348956

**Published:** 2026-05-11

**Authors:** Sujata Kandel, Hari Prasad Kaphle

**Affiliations:** School of Health and Allied Sciences, Pokhara University, Pokhara, Nepal; Drake University College of Pharmacy and Heath Sciences, UNITED STATES OF AMERICA

## Abstract

**Background:**

Understanding nurses’ engagement and drivers is crucial as it impacts patient experience, safety and healthcare quality. Despite the rapid growth of the private healthcare sector and numbers of nurses, little is known about the factors influencing work engagement and its relationship with job satisfaction and turnover intention among nurses in private hospitals of Nepal, where working conditions and organizational structures differ from the public sector. This study aims to assess work engagement levels, identify factors that increase or decrease work engagement among nurses, and determine its association with job satisfaction and turnover intention.

**Methods:**

A facility-based cross-sectional study among 211 nurses in private hospitals of Pokhara was conducted from September to October, 2024 using a self-administered structured questionnaire. The questionnaire assessed work engagement as the dependent variable and job satisfaction and turnover intention as outcome variables. Work engagement was measured using the 17-item Utrecht Work Engagement Scale, while job satisfaction and turnover intention were measured using the 5-item Short Index of Job Satisfaction and the 3-item Turnover Intention Scale, respectively. Multistage random sampling was applied, and data were entered in Epi Data and analyzed in SPSS. Descriptive statistics and inferential analyses, including Chi-square tests was used to identify the associated factors and logistic regression was used to measure the strength of association between opportunities for career development and perceived workload with work engagement.

**Results:**

The mean score for work engagement among nurses working in private hospitals is 4.29 ± 0.72, which is considered to be high. Binary logistic regression showed that opportunities for career development (aOR: 9.256, 95% CI: 1.803–47.511) and perceived workload (aOR: 3.586, 95% CI: 1.170–10.989) were found to be associated with work engagement. Similarly, there found a significant association between work engagement and job satisfaction (aOR: 5.076, 95% CI: 2.557–10.076).

**Conclusion:**

Nearly two third of the nurses working in private hospitals had high work engagement level. In multi-variates logistic regression analysis, opportunities for career development and perceived workload were found to be associated with work engagement. Work engagement was found to be significantly and positively associated with job satisfaction.

## Introduction

The World Health Organization views nurses as the backbone of health care system [1]. An engaged and dedicated nursing staff is necessary for providing safe and high-quality patient care which significantly predicts patient outcomes, including reduced mortality rates [2–4].

Work engagement is a positive, fulfilling, work-related state of mind that is characterized by vigour, dedication, and absorption [5]. It is a positive state opposing burnout, associated with higher organizational commitment, job satisfaction, and lower turnover intentions [6,7]. Work engagement among healthcare professionals is essential for achieving excellence in healthcare, especially in nursing, a profession grounded in compassion and humanity [8]. Higher work engagement among nurses promotes retention and fosters behaviors that strengthen patient outcomes, interprofessional collaboration and overall organizational performance [2]. Despite this evidence, global reports highlight alarming nursing shortages of 5.9 million as a result of turnover [9]. The shortage of nurses is adding burden to the present workforce, increasing the workload [10] which significantly undermines progress towards health-related SDGs, particularly in LMIS where workforce constraints are more severe, and retention is increasingly difficult [11]. Nepal is also facing severe shortage with only 9,951 nurses actively working out of 14,099 vacancies [12]. According to data from Nepal Nursing Council, the nurse-to-patient ratio is 2.10:1,000 which is below WHO recommendations, exacerbating the strain on healthcare services [13]. Many nurses from Nepal travel to foreign countries for better employment opportunities [14]. Lack of career development opportunities, low salary are associated with brain drain among Nepalese nurses [15], highlighting the urgent need to address workforce challenges to improve healthcare delivery [16].

A range of factors have been identified in the literature as influential for work engagement. Individual characteristics such as years of experience, marital status, health condition, position at work have been shown to affect engagement levels [17]. Similarly, supervisor support, social support, peer support, opportunities for career growth, reward and recognition positively associated with work engagement [18–20]. Inversely, workload, poor teamwork practice, high burnout, lack of resources has been identified as a negative factor influencing nurses’ work engagement [21,22]. Understanding these factors is crucial as increased work engagement leads to favourable outcomes like job satisfaction and the negative result in employees intent to leave [23].

“Job satisfaction” describes the way individuals behave and feel about their jobs. It is the extent to which employees like their jobs [24]. Job satisfaction is closely associated with engagement, with higher engagement promoting better performance and high level of satisfaction [23]. Research has shown that social aspects of job are significant predictor of job satisfaction whereas, organizational issues, workload, and unfavourable working conditions are strongly associated with job dissatisfaction [25,26].

Turnover intention is the desire of an individual to leave their current job and search for other opportunities; it is often a reliable predictor of actual turnover behaviour [27]. It exacerbates healthcare shortages and negatively impacts patient safety, care quality, and outcomes [28,29].Healthcare organizations have consistently faced the issues of high nurse turnover rates,highlighting the need to identify the factors that can help lower turnover rates, enhance job satisfaction and maintain high patient service quality [30].

In Nepal, private hospitals have emerged rapidly as a complement to the public system, particularly in urban centers like Pokhara, driven by growth in demand for private healthcare, rising out-of-pocket expenditure, and shifting policy emphasis on public-private collaboration [31,32]. The private sector delivers a substantial share of curative service and healthcare to about 70% of the population [33], employing a large proportion of nursing workforce. The evolving private healthcare sector influences nurses work conditions, resources, and organizational dynamics, shaping their work engagement, job satisfaction and turnover intention.

Studies on work engagement among nurses in private hospitals remains limited, even though private providers increasingly deliver a substantial share of curative services in LMICs like Nepal. This contextual gap is important because private hospitals operate under managerial practices, reward systems and workload pressures that often differ from public institution and therefore may shape engagement in distinct ways. Some recent studies highlighted the need to foster work environments that enhance engagement, job satisfaction, and retention [34]. Greater work engagement among nurses benefits both healthcare organizations and patients by improving productivity, job satisfaction, and care quality while reducing burnout and turnover. Therefore, this study aims to assess work engagement levels, identify factors that increase or decrease work engagement among nurses, and determine its association with job satisfaction and turnover intention.

## Methodology

### Study design and study setting

A facility-based cross-sectional study was conducted in the private hospitals of Pokhara from September 1 to October 1, 2024. Pokhara, a metropolitan city located in central Nepal, is the capital of Gandaki Province and lies approximately 200 kilometers west of Kathmandu, the capital city of Nepal. There are a total of 44 hospitals in Pokhara, of which 40 are private hospitals. Among these, two hospitals have more than 300 beds, while the remaining 38 hospitals have fewer than 300 beds.

### Study population

All the registered nurses working in private hospitals of Pokhara were the study population. Nurses with work experience of at least 6 months and those who were volunteered to participate in the study were included while, nurses who were absent during data collection period and those who were not willing to participate were excluded.

### Sample size and sampling technique

The sample size was calculated using formula n = z^2^pq/d^2^ where, Z = value of standard normal distribution at a level of significance with 95% of C.I (z)= 1.96, prevalence (p)=0.59 [20], margin of error (d)=0.05. Thus, the final sample size was 211 after adjusting 10% non-response rate.

A multistage sampling technique was employed to ensure a representative sample of nurses from private hospitals in Pokhara. In the first stage, 14 out of 42 hospitals were selected using simple random sampling (SRS). In the second stage, nurses from each hospital were proportionally assigned based on staff size. Lastly, the individual participants from each hospital were randomly selected using lottery method.

### Study variables

The dependent variable of this study was work engagement, while independent variables were divided into 3 parts, i.e., personal, organizational and job-related factors. The outcome variables were job satisfaction and turnover intention.

Work engagement was assessed using the Utrecht Work Engagement Scale (UWES)-17 developed by Schaufeli et al. It consists of 17 items across 3 subscales: vigor (6 items), absorption (6 items) and dedication (5 items). Responses were recorded on a 7-point Likert scale to rate their frequency of feeling certain ways about their work, ranging from 0 (never) to 6 (always) [35]. To determine overall reported work engagement, each nurse’s total UWES score were calculated, with higher scores indicating higher engagement. The cutoff values were statistically established to provide clear meaning for the derived scores for high (4–6), moderate (2 < x < 4), and low (0 < x < 2) reported engagement [36].

The job satisfaction among nurses was measured using Short Index of Job Satisfaction (SIJS) which have 5 items. For each item, respondents responded on a 5-point Likert scale with the endpoints 1 = strongly disagree to 5 = strongly agree. The internal consistency reliability of the tool reported in Brayfield and Rothe (1951) was.75. The analysis was done by calculating mean and standard deviation of each question and taking average of it [37].

Turnover intention among nurses was measured using three item scale developed by Michaels and Spector 1982 [38]. The internal consistency reliability reported was 0.93. Respondents responded on a 5-point Likert scale with 1 = strongly disagree to 5 = strongly agree [39]. It was analyzed by computing the mean and standard deviation for each item and then calculating the composite mean score for the overall scale.

### Data collection tools and techniques

The structured self-administered questionnaire was used for data collection by adapting from reviewing pieces of literature. The scale was translated and improved under the close supervision of the research supervisor. To make sure that all technical words were appropriate for the target culture and language, the supervisor took part in the expert evaluation. This oversight ensured the instrument’s reliability before moving to the pre-testing phase. Pretesting of tools was done in 10% of the total sample size (n = 21) from a hospital of similar setting. A necessary modification was made based on the pretest findings in the data collection tool consulting with the supervisor and subject expert. The internal consistency reliability of the instrument (work engagement) for this study was 0.811, job satisfaction was 0.631 and turnover intention was 0.651.

With official access approval, the data was collected during morning and evening shifts, and on-site coordination allowed for a 95% response rate. Although conducting surveys during working hours guaranteed active participation, it might have added biases due to social preferences or time constraints. The research supervisor kept an eye on the procedure to guarantee confidentiality of the participants and response accuracy in order to preserve data integrity.

### Data management and analysis

Collected data were entered into Epi-Data version 3.1 and the data was cleaned and exported to Statistical Package for Social Sciences (SPSS) version 26 for analysis. Negatively worded items were reverse scored. The data was summarized in terms of frequency, percentage for categorical variable, mean and SD for descriptive analysis of both dependent and independent variables. Then, chi-square test (p-value <0.05 at 95% C.I) was used for testing for association between the dependent and independent variables. We applied binary logistic regression to model the associations between independent variables and dependent variable (work engagement), and providing adjusted odds ratios to quantify these relationships in binary logistic regression, we incorporated all variables with a chi-square test p-value below 0.05. Unadjusted and adjusted odds ratio (OR) at 95% level of confidence was calculated.

### Ethical Approval

Ethical Approval was taken from the Institutional Review Committee (IRC) of Pokhara University (Reference Number: 105/2081/82, approval date: August 30, 2024. Similarly, permission was taken from the hospitals administration before the study. Written informed consent was taken from the nurses participating in the data collection and were informed about their right to withdraw from the study at any time without providing a reason. Confidentiality of data and privacy of information obtained from the study participants was maintained throughout the study.

## Results

### Baseline characteristics of participants

A total of 211 nurses participated in the study. The mean age of participants was 24.64 ± 4.518. Most of the participants (85.3%) were working as staff nurse. Majority of participants had < 4 years of professional experience (70.1%).

Regarding organizational factors, above half of the participants found the organization and supervisor to be moderately supportive. Opportunities for career development were rated as very sufficient by one third participants (34.6%). Most of the participants reported not receiving rewards (75.4%).

Regarding job-related characteristics, participants worked across various units, most commonly general (27.5%), medical 23.2% and 20.9% in critical care units. A large majority of participants (88.2%) worked both day and night shifts. Most participants (74.9%) worked ≤ 8 hours per day and ≤ 48 hours per week (90%). Workload was generally perceived as moderate by majority of participants (80.1%). (Table 1)

**Table 1:** Baseline characteristics of participants.

Variables (n = 211)	Frequency (n)	Percentage (%)
**Sociodemographic factors**		
**Age of participants**	**(Mean = 24.64, SD = 4.518)**	
≤ 25 years	144	68.2
> 25 years	67	31.8
**Marital status of participants**		
Married	61	28.9
Unmarried	150	71.1
**Highest educational qualification**		
SEE/SLC	21	10.0
PCL	154	73.0
Bachelor’s	36	17.0
**Nursing qualification**		
Technical SLC/ANM	21	10.0
PCL nursing	154	73.0
B Sc. Nursing/ BN	36	17.0
**Position at work**		
ANM	21	10.0
Staff nurse	180	85.3
Nursing Officer	7	3.3
Matron	3	1.4
**Work experience in nursing field**	**(Mean = 3.81, SD = 3.862)**	
< 4 years	148	70.1
≥ 4 years	63	29.9
**Work experience in the same hospital**	**(Mean = 2.07, SD = 2.040)**	
≤ 2 years	161	76.3
>2 years	50	23.7
**Perceived health condition**		
Poor	1	0.5
Good	157	74.4
Very good	53	25.1
**Sleep length**	**(Mean = 7.32, SD = 0.966)**	
<8 hours	193	91.5
≥8 hours	18	8.5
**Organizational factors related information**		
**Perceived organizational support**		
Not supportive at all	35	16.6
Moderately supportive	110	52.1
Very supportive	66	31.3
**Perceived supervisor support**		
Not supportive at all	9	4.2
Moderately supportive	116	55.0
Very supportive	86	40.8
**Team and co-worker support**		
Not supportive at all	0	0.0
Moderately supportive	94	44.5
Very supportive	117	55.5
**Opportunities for career development**		
Not sufficient at all	40	19.0
Somewhat sufficient	98	46.4
Very sufficient	73	34.6
**Receive reward**		
Yes	52	24.6
No	159	75.4
**Receive recognition**		
Not at all	57	27.0
Sometimes	135	64.0
Most of the times	19	9.0
**Job related information**		
**Type of work setting**		
Emergency	19	9.0
General	58	27.5
Medical	49	23.2
Surgical	17	8.1
Critical care unit	44	20.9
All unit	24	11.4
**Work shift pattern**		
Day shift	25	11.8
Night shift	0	0.0
Both shift	186	88.2
**Total night shifts in a month**	**(Mean = 8.62, SD = 1.455)**	
≤ 8 times	157	84.4
> 8 times	29	15.6
**Working hours per day**	**(Mean = 7.78, SD = 2.539)**	
≤ 8 hours	158	74.9
> 8 hours	53	25.1
**Working hours per week**	**(Mean = 48.23, SD = 4.492)**	
≤ 48 hours	190	90
> 48 hours	21	10
**Perceived workload**		
Light	24	11.4
Moderate	169	80.1
Heavy	18	8.5

### Work engagement level of participants across its dimension

Overall, about two-third of participants (63%) reported high work engagement. Dedication scored highest among the dimensions, followed by absorption and vigour. (Table 2)

**Table 2:** Work Engagement level of participants with its dimension.

	Vigor n(%)	Dedication n(%)	Absorption n(%)	Total work engagement n(%)
**Low**	6 (2.8)	3 (1.4)	3 (1.4)	1 (0.5)
**Moderate**	109 (51.7)	23(10.9)	79 (37.4)	77 (36.5)
**High**	96 (45.5)	185 (87.7)	129 (61.1)	133 (63.0)

### Mean and standard deviation of work engagement and its dimension

The total mean score and standard deviation of work engagement in our study was 4.29 ± 0.72. Vigour got the lowest mean score of followed by absorption and dedication had the highest mean score (Table 3).

**Table 3:** Mean and Standard deviation of Work Engagement and its Dimension.

Work engagement	Mean score	Standard deviation	Min-Max
**Total score**	4.29	0.72	1.06-5.53
**Vigor**	3.85	0.97	1.00-5.67
**Dedication**	4.92	0.87	0.60-6.00
**Absorption**	4.19	0.86	1.17-5.67

### Job satisfaction among nurses

Regarding job satisfaction among nurses working in private hospitals nearly two-thirds (62.1%) of participants reported low job satisfaction, while just over a third (37.9%) reported high job satisfaction. (Table 4)

**Table 4:** Job satisfaction among nurses.

Job satisfaction	Frequency	Percentage
Low job satisfaction	131	62.1%
High job satisfaction	80	37.9%

### Turnover intention among nurses

Regarding turnover intention among nurses, more than half (55.5%) of participants reported low turnover intention, whereas nearly half (44.5%) had high turnover intention (Table 5).

**Table 5:** Turnover intention among nurses.

Turnover intention	Frequency	Percentage
Low turnover intention	117	55.5%
High turnover intention	94	44.5%

### Factors associated with work engagement among nurses from multi-variable logistic regression

The participants with sufficient opportunities for career development (aOR: 9.256, 95% CI: 1.803–47.511) were 9 times more likely to be engaged in their work compared to those without opportunities for career development. Although the large confidence interval (1.803–47.511) suggests poor precision due to possible sample size limits within specific strata, the high aOR (9.256) indicates a robust correlation. Therefore, the point estimate should be considered as a directional indicator rather than a precise size of effect, even though the results demonstrate a significant positive link. Similarly, participants who perceived their workload light (aOR: 3.586, 95% CI: 1.170–10.989) were 4 times more likely to be engaged in their work compared to those who perceived their workload as heavy (Table 6).

**Table 6:** Factors associated with work engagement among nurses from multi-variate logistic regression.

Variables (n = 211)	Unadjusted		Adjusted	
	OR (95% CI)	p-value	OR (95% CI)	p-value
**Personal factors**				
**Age**				
< 25	Ref	–	Ref	–
≥ 25	1.944 (1.032-3.664)	0.040*	1.205(0.497-2.924)	0.680
**Work experience in nursing field**				
< 4 years	Ref	–	Ref	–
≥ 4 years	2.118 (1.101-4.075)	0.025*	1.330(0.511-3.464)	0.559
**Work experience in same hospital**				
≤ 2 years	Ref	–	Ref	–
>2 years	2.200 (1.070-4.524)	0.032*	1.192 (0.474-2.997)	0.709
**Organizational factors**				
**Perceived organizational support**				
Not supportive at all	Ref	–	Ref	–
Moderately supportive	1.529 (0.712-3.284)	0.276	0.456 (0.098-2.121)	0.317
Very supportive	3.600 (1.496-8.661)	0.004*	0.474 (0.081-2.787)	0.409
**Perceived supervisor support**				
Not supportive at all	Ref	–	Ref	–
Moderately supportive	1.020 (0.260-3.992)	0.978	0.893 (0.189-4.216)	0.887
Very supportive	2.191 (0.541-8.876)	0.272	1.727 (0.350-8.519)	0.502
**Opportunities for career development**				
Not sufficient at all	Ref	–	Ref	–
Somewhat sufficient	2.047 (0.971-4.316)	0.060	5.024 (1.146-22.029)	0.032**
Very sufficient	4.820 (2.087-11.130)	<0.001*	9.256 (1.803-47.511)	0.008**
**Job related factors**				
**Work shift pattern**		–		–
Day shift	3.469 (1.144-10.513)	0.028*	1.755 (0.496-6.212)	0.383
Both shift	Ref		Ref	
**Working hours per day**				
≤ 8 hours	2.179 (1.156-4.105)	0.016*	1.909 (0.932-3.913)	0.077
>8 hours	Ref	–	Ref	–
**Perceived workload**				
Light	3.729 (1.332-10.442)	0.012*	3.586 (1.170-10.989)	0.025**
Moderate	4.857 (1.301-18.132)	0.019*	3.488 (0.807-15.084)	0.094
Heavy	Ref	–	Ref	–

* Significant at p < 0.05 from bivariable binary logistic analysis.

** Significant at p < 0.05 from multivariable logistic analysis.

### Association of work engagement with job satisfaction and turnover intention

From binary logistic analysis, there found participants with high work engagement were 5 times more likely to be satisfied with their job as compared to low work engagement (aOR: 5.076, 95% CI 2.557–10.076) and participants with low work engagement were 2 times more likely to had turnover intention with their job as compared to high work engagement (aOR: 1.975, 95% CI 1.121–3.482) (Table 7).

**Table 7:** Association of work engagement with job satisfaction and turnover intention.

Variables	Unadjusted	
	OR (95% CI)	p-value
**Work engagement with job satisfaction**		
Low work engagement	Ref	
High work engagement	5.076 (2.557-10.076)	<0.001*
**Work engagement with turnover intention**		
Low work engagement	1.975 (1.121-3.482)	0.019*
High work engagement	Ref	

* Significant at p < 0.05 from binary logistic analysis.

### Association of work engagement with turnover intention

From binary logistic regression there found a significant association between work engagement and turnover intention (aOR: 1.975, 95% CI 1.121–3.482) where participants with low work engagement were 2 times more likely to had turnover intention with their job as compared to high work engagement.

## Discussion

This study assessed the level of work engagement and its associated factors among nurses working in private hospitals of Pokhara, Nepal. It also determined the association of nurse’s work engagement with job satisfaction and turnover intention.

The mean of work engagement score indicated a high level of engagement among participants in this study findings which is consistent with the studies from China [40], Eygpt [41], USA [42], Spain [43]. These findings suggests that work engagement among nurses remains fairly consistent across countries, despite variation in working environment. The high levels of work engagement in these studies can be attributed to organizational support, opportunities for career development, intrinsic motivation of nurses and better working condition. Such a high level of work engagement may reflect that committed nurses feel energetic and absorbed in their work and enhance their sense of meaning and self-realization [36].

Here, the study shows that 62.1% low job satisfaction and 44.5% high turnover intention despite high engagement which is contradictory findings. This findings highlight a crucial paradox in the private healthcare industry: a highly engaged staff that is nevertheless unhappy and intent to leave. Although a strong professional ethos is shown by the high level of engagement, the dissatisfaction and high turnover intention rate underscores potential impact of effort-reward imbalance, poor work-life balance, better external opportunities.

In contrast, the findings in this study was higher than studies conducted among nurses in Beijing, China [20], Japan [18], United states [23]. The possible reason for the variation in China could be due to high workload and limited organizational support, factors that tend to suppress work engagement. The possible justification for the variation in Japan could be due to different nursing specialty, timing, measurement approaches and working environment. The discrepancy with United states might be due to burnout and stress, especially following the COVID-19 pandemic, resulting in engagement scores frequently lower than those observed in this study.

The findings of the study showed an association between opportunities for career development and work engagement. Nurses who had got sufficient opportunities for their professional growth were substantially more engaged in their work. The finding was supported by a study conducted in Australia where opportunities for career growth is significantly associated with work engagement (β = 0.18, p < .05) [44]. A study from Columbia found significant and positive association between opportunities for professional development and work engagement (r = 0.19, p < 0.01) [45], with an even stronger effect when combined with colleague support. The findings was supported by a study done in Kenya’s public health sector (r = 0.670, p < 0.01) [46], South African nurses in 2020 (β = 0.16, p < 0.001) [47]. A study from Saudi Arabia affirmed that opportunities for career development promotes a positive and supportive work environment within organization that enhance work engagement [48].These findings underscore the importance of investing in structured career development initiatives, such as mentorship programs, skill-enhancement training, and clear pathways for professional growth. Such initiatives can not only enhance individual nurse engagement but also contribute to organizational effectiveness by reducing turnover and improving job satisfaction.

Similarly, workload also showed a significant relationship with work engagement. The findings from this study showed that nurses who perceived their workload as light were more likely to be engaged in their work than those who perceived their workload as heavy. This aligns with the evidence from Nigeria (β=−.23, p < .01) [49] and China (βdirect = −0.353, p < .001) [40] which suggests that excessive job demands reduce nurses’ ability to remain energetic and committed to their roles. In high-pressure healthcare environment, sustained workload stress may limit opportunities for recovery and reflection, ultimately weakening engagement and overall performance. These findings emphasize the need for balanced staffing, supportive management and workload distribution strategies to maintain nurses engagement.

In contrast, workload levels are linked to increased engagement at work according to the study among acute care nurses from United States(β = .30, p < .001) [50]. The variation in the study findings may be attributed to difference in organizational culture and support systems.

This study found that nurses with high work engagement level were significantly more likely to be satisfied with their jobs compared to those with low work engagement. Similar findings have been reported in studies from the United states(P < 0.001) [23] and China (r = 0.519, p < 0.05) [51]. Similarly, a study of engineers working in Egyptian oil and gas company reported a significant association between job satisfaction and work engagement (*r* = 0.396, *p* < 0.05) [52]. Employees that are engaged are emotionally and cognitively immersed in their jobs, which increases motivation, enthusiasm, and a sense of accomplishment. A work environment that encourages engagement—through organizational support, autonomy, and growth opportunities—generates job satisfaction by instilling a feeling of purpose and connection, which improves retention, performance, and overall well-being.

In this study, nurses with low work engagement were more likely to report high turnover intention. Evidence from Ghana (β = −0.0367, p < 0.05) [11] and China (β = −0.283, p < 0.001) [27] also revealed the negative correlation between turnover intention and work engagement among nurses. Another study from China also supports this inverse relationship (β = −0.13, *p* < 0.001) [53]. Employees who are highly engaged demonstrate greater energy and enthusiasm at work, which enhances their sense of self-worth and strengthens their intention to stay. Organization that aims to improve nurse retention rates should focus on enhancing their work engagement. This can be achieved through providing them the opportunities for career development, fostering the suitable work environment, ensuring a healthy work life balance.

It is important to note that this study has a number of limitations. First, establishing a causal relationship between independent and dependent variables is impossible due to the cross-sectional character of the study design. Second, participants may respond biasedly as self-administered questionnaires are used to assess the variables being studied. As the research was conducted only among nurses working in the private hospitals, this may limit the generalizability of the study findings in other settings, i.e., government hospital and to the wider scale because of different organizational culture.

## Conclusion

This study concludes that nurses working in private hospitals of Pokhara had high levels of work engagement with nearly two third of the nurses were highly engaged. When the subscales were evaluated separately, vigour got the lowest mean score, while the dedication had the highest mean score. Opportunities for career development and perceived workload were significantly associated with work engagement. The two outcome variables, i.e., job satisfaction and turnover intention showed association with work engagement in bi-variate analysis.

The hospital management should focus on career development opportunities of nurses to enhance their work engagement. The management can implement flexible working schedule to reduce the perceived workload among nurses that can hinder their work engagement level. Maintain an environment of respect, collaboration, and open communication at work among team members and supervisors is also important. Furthermore, mixed-method studies should be conducted among all health care workers, across different hospital setting, as this will help enhance work engagement by identifying the associated factors and allow for meaningful comparison across contexts.
